# Modulating Vaccinia Virus Immunomodulators to Improve Immunological Memory

**DOI:** 10.3390/v10030101

**Published:** 2018-02-28

**Authors:** Jonas D. Albarnaz, Alice A. Torres, Geoffrey L. Smith

**Affiliations:** Department of Pathology, University of Cambridge, Tennis Court Road, Cambridge CB2 1QP, UK; jd732@cam.ac.uk (J.D.A.); at678@cam.ac.uk (A.A.T.)

**Keywords:** vaccinia virus, vaccine, smallpox, immune evasion, genetic engineering, immunological memory, orthopoxviruses

## Abstract

The increasing frequency of monkeypox virus infections, new outbreaks of other zoonotic orthopoxviruses and concern about the re-emergence of smallpox have prompted research into developing antiviral drugs and better vaccines against these viruses. This article considers the genetic engineering of vaccinia virus (VACV) to enhance vaccine immunogenicity and safety. The virulence, immunogenicity and protective efficacy of VACV strains engineered to lack specific immunomodulatory or host range proteins are described. The ultimate goal is to develop safer and more immunogenic VACV vaccines that induce long-lasting immunological memory.

## 1. Introduction

The development of vaccines and subsequent widespread vaccination has resulted in the eradication of two virus diseases, smallpox [1] and rinderpest [2], the control of several others and, together with other factors such as improved hygiene, has led to improved animal welfare and an increase in human life expectancy. Scientific research has provided vaccines against several other diseases that could be eradicated (measles, mumps and rubella (MMR), for instance) but there has been insufficient will to ensure adequate vaccine usage to achieve eradication. For example, despite the live attenuated vaccine against measles having been available for more than 50 years and its use having greatly decreased the incidence of measles and prevented an estimated 20 million deaths between 2000 and 2015 [3,4], the disease remains and is resurgent due to under-utilisation of the vaccine. This under-utilisation has been fuelled by the erroneous claim that MMR causes autism, the increased activity of anti-vaccination groups, the decreased concern about the disease as it became less common and political apathy. In consequence, there have been serious outbreaks of measles in several countries including USA (Minnesota, 2017) [5], England and Wales (more than 2000 cases in 2012 and the highest level for 2 decades) [6,7,8], other parts of Europe [9] and Latin America [10]. This highlights the importance of maintaining vaccination to prevent the dissemination of infectious diseases. The eradication of measles and some other infectious diseases remains an achievable goal and we should be inspired by the eradication of both rinderpest and smallpox.

Smallpox was a greatly feared infectious disease that caused hundreds of millions of deaths since it was first reported approximately >3000 years ago [11]. Indeed, in the 20th century alone, more than 100 years after an effective vaccine was available, smallpox killed an estimated 400 million people. Smallpox was caused by *Variola virus* (VARV), a member of the *Orthopoxvirus* genus of the *Poxviridae* [12]. Unlike several other orthopoxviruses, such as cowpox virus (CPXV) and monkeypox virus (MPXV) that infect a wide range of mammals, VARV was restricted to a single species, *Homo sapiens* and this restriction contributed greatly to smallpox eradication. An ancient strategy to prevent smallpox was “variolation” or “inoculation” that consisted of the deliberate infection of people, usually in the arm, with infectious material taken from pustules from a prior case of smallpox. This practice was introduced into Europe by Mary Wortley Montagu, who observed this practice among the Turks in Constantinople in 1717. This procedure was dangerous and was associated with a fatality rate of 0.5–2% (due to smallpox), yet it was used because of the much greater fatality rate of natural smallpox (20–30%) [11,13]. Variolation prevented natural cases of smallpox, but it also enabled VARV to spread from variolated people and contributed to smallpox outbreaks [11]. Nonetheless, it was the only means to prevent smallpox until 1796 when Edward Jenner introduced vaccination. Jenner and others had observed that milkmaids who were infected by CPXV acquired a local mild disease and subsequently were resistant to smallpox [14]. Jenner was credited with the invention of vaccination because he set out to test experimentally the hypothesis that it was the prior infection with cowpox that prevented subsequent smallpox. Jenner first vaccinated a boy, James Phipps, with cowpox material taken from the hand of a milkmaid, Sarah Nelmes, and a few weeks later challenged Phipps with VARV (variolation). The child was resistant to variolation and, after repeating the experiment in several other children, Jenner published his observation privately in 1798 [14]. Jenner was convinced of the effectiveness of this practice and in 1801 he predicted the eradication of smallpox saying, “It now becomes too manifest to admit of controversy that the annihilation of smallpox, the most dreadful scourge of the human species, must be the final result of this practice” [15].

The process Jenner introduced became known as “vaccination” and the agent used as a “vaccine” after the Latin word for cow, “vacca,” for reviews see [16,17]. The principle behind the efficacy of vaccination against smallpox, unknown to Jenner, relies on the antigenic conservation and cross-protective nature of orthopoxviruses, such that prior infection by one orthopoxvirus provides protection against subsequent infection by any other orthopoxvirus [11,18]. During the 19th century, Jenner’s vaccine against smallpox was spread around the world but it was not until 1959 that the World Health Assembly adopted a resolution to achieve the global eradication of smallpox. In 1967 an intensified smallpox eradication campaign began that utilised surveillance, quarantine and ring vaccination. This was highly effective and in just 10 years resulted in the global eradication of smallpox. The last naturally occurring case was in Somalia in 1977 and after a further 2 years of surveillance the WHO certified eradication was complete [1] thereby fulfilling Jenner’s prophecy. Several factors contributed to the success of this programme. These included the fact that VARV infected only humans, it did not establish latent or persistent infections, the disease was easily recognisable, the causative agent was genetically stable and did not escape existing immunity by antigenic variation and the vaccine was plentiful, cheap, stable and easy to administer. Most important there was widespread enthusiasm and drive to see the job completed.

## 2. Continuing Threat of Smallpox and Other Zoonotic Orthopoxviruses

### 2.1. Remaining VARV Stocks

Despite eradication of the disease smallpox, the causative agent, VARV, remains in two high security laboratories: one in the State Research Centre of Virology and Biotechnology (Vector) in the Russian Federation and one at the Centers for Disease Control and Prevention (CDC) in the USA. Following the eradication of smallpox, all known stocks of VARV were catalogued and centralised in these maximum-security laboratories. Thereafter, the WHO asked the WHO Ad Hoc Committee on Orthopoxviruses to consider what should happen to the remaining VARV stocks. After debate, the Committee recommended in 1994 that all remaining stocks of VARV should be destroyed. This recommendation was adopted by the World Health Assembly in 1996 and the destruction scheduled for 1999. In the meantime, limited work with VARV in these two high security laboratories was permitted to enable essential research for public health benefit. This work was limited to the development of diagnostic tests for cases of smallpox, antiviral drugs that could treat smallpox and a safer vaccine that would be tolerated by those in whom use of the conventional smallpox vaccine was contraindicated. To oversee this research the WHO set up the Advisory Committee on Variola Virus Research (ACVVR). This committee has met annually since 1999 and reported on progress towards the research objectives for 19 consecutive years and, although considerable progress has been made, the work is considered incomplete and the VARV stocks have not been destroyed.

### 2.2. Recreation of Orthopoxviruses by Synthetic Biology

While temporary retention of VARV has been permitted, an additional complication has arisen, namely methods to recreate an orthopoxvirus from chemicals using synthetic biology. Since 2001 it has been possible to recover a live orthopoxvirus from non-infectious DNA by the introduction of this DNA into cells that are then infected by a different poxvirus [19]. Recently, this was repeated with horsepox virus (HSPV) but in this case the virus was recreated by the synthesis of virus genome fragments from chemicals, rather than by using existing cloned DNA, followed by the introduction of these fragments into cells [20]. These cells were then infected with another (helper) poxvirus and live HSPV was recovered from these cells. The genome sequences of about 50 strains of VARV have been determined and are in the public domain [21,22]. What is possible for one orthopoxvirus will be possible for others, so there is little doubt that live VARV could be recreated from simple chemicals. This has never been done, it would take a sustained and deliberate effort and is prohibited absolutely by the WHO. Also prohibited is any genetic engineering with VARV, the holding of more than 20% of a VARV genome in any laboratory outside the 2 high security laboratories permitted to hold VARV and the introduction of any VARV DNA into any laboratory that holds any other live orthopoxvirus [23]. These restrictions are designed to eliminate any possibility that VARV, or a virus with VARV-like properties, could be recreated accidentally by recombination between VARV DNA and another orthopoxvirus genome inside living cells.

The possible deliberate use of VARV in bioterrorism has been of heightened concern after the 9/11 terrorist attacks in USA, for if terrorists were willing to commit mass murder by flying aeroplanes into densely populated buildings, would they hesitate to release VARV if they had access to it [24]? Further, there are concerns about unofficial stocks of VARV that have been retained accidentally or deliberately [25]. The discovery of vials containing infectious VARV at the National Institutes of Health (NIH), Bethesda, USA in 2014 highlighted this issue [26]. There is also concern that infectious VARV might be present within corpses of victims of smallpox that have been buried in permafrost. Although no infectious VARV has been found in buried smallpox victims, fragmented pieces of VARV DNA are present in such material and it has been possible by mass DNA sequencing and computer-mediated DNA analysis to deduce a full VARV genome sequence from a mummified corpse of a VARV victim who died in Vilnius, Lithuania, around 1650 [27].

Arguments for the destruction of VARV stocks have included: (i) destruction being the natural conclusion of the smallpox eradication campaign; (ii) the removal of the risk of virus escape from these laboratories; and (iii) the elimination of the costs associated with storing the virus safely and securely [24]. The principal argument against the destruction of all VARV stocks now is the possibility that live virus might be needed to evaluate new drugs against smallpox [28,29].

### 2.3. Emergence and Re-Emergence of Orthopoxviruses

As smallpox was eliminated country by country, smallpox vaccination declined and was stopped after eradication was certified in 1979. Indeed, routine vaccination against smallpox was discontinued in many countries prior to eradication. Consequently, very few people are vaccinated nowadays so that a large proportion of the world’s population is unvaccinated and therefore potentially at risk from VARV and other orthopoxviruses [30]. At present, infections of humans by zoonotic orthopoxviruses are increasing. Of these, MPXV is of greatest concern and causes a severe, smallpox-like disease in humans, with a mortality rate of 1–8% [31]. Although monkeypox has occurred mostly in central Africa such as in the Democratic Republic of the Congo (DRC), cases have been reported in other African countries and, in 2003, a zoonotic outbreak occurred in the USA, caused by the importation of MPXV-infected rodents from West Africa [32]. In 2017, there were outbreaks of monkeypox in several African nations including Nigeria, DRC and Republique Centre Africaine (RCA) [33]. Furthermore, cases of human monkeypox have increased in DRC over the last 30 years and one contributory factor is the increasing proportion of the population that is immunologically naïve to orthopoxviruses following cessation of smallpox vaccination [34]. Another factor that enhances the likelihood of orthopoxvirus infections in humans is the human immunodeficiency virus (HIV) epidemic and the consequential acquired immune deficiency syndrome (AIDS). Viruses that are prevented from becoming established in humans by a fully functional immune system have increased opportunity to infect and spread in those with an impaired immune system and thereafter to adapt and acquire human-to-human transmission.

In Europe in the last two decades, there have been increased CPXV outbreaks, some resulting from transmission from pet animals to humans [35,36,37,38]. Although infections are a sporadic cause of disease in domestic animals [39,40] and humans [41], CPXV can also cause severe and sometimes fatal disease in immunosuppressed individuals [42,43,44].

Lastly, zoonotic vaccinia virus (VACV) outbreaks have been described in Asia and South America [30]. The clinical presentation of VACV infection in humans is usually associated with localised exanthematous lesions but systemic clinical manifestations can also occur [45]. In India, buffalopox (caused by a VACV strain) outbreaks have been reported since 1934, but outbreaks of buffalopox have also been reported in Nepal, Pakistan, Indonesia and Egypt. Buffalopox affects mainly buffaloes, though infection of humans has also been recorded [46,47]. In Brazil, VACV circulation was first reported in the 1960s and 1970s, however the first outbreak involving cattle and humans was only reported in 1999 [45,48]. Thereafter, several other outbreaks were reported, affecting mainly bovines, equines, rodents and humans [49,50,51,52]. The extent of VACV circulation in Brazil is uncertain because of misdiagnosis and underreporting [45]. Moreover, evidence of VACV circulation was also found in Amazon monkeys and semi-domestic rodents, suggesting the virus is circulating in the wild [53,54]. More recently, VACV circulation has also been detected in cattle from Argentina, Uruguay and Colombia [55,56,57].

Emergence of novel orthopoxviruses also poses a threat to an increasingly immunologically naïve population, as shown by the recent cases in humans in Georgia and in Alaska, USA [58,59]. Both reports highlight the zoonotic nature of these infections, as was an outbreak of an uncharacterised orthopoxvirus in macaques in an Italian primate sanctuary [60].

## 3. Vaccine Development against Smallpox and Other Orthopoxviruses

Initially, smallpox vaccine material was obtained from lesions of cows presumably infected with CPXV and live vaccine was retained by arm-to-arm vaccination in humans or by skin scarification in animals, such as bovine calves [11]. In 1939, the smallpox vaccines being used in the 20th century were shown to be distinct from CPXV and were termed VACV [61]. So, if Jenner used CPXV in 1796 (and this is not certain given the virus is no longer available for analysis), sometime between 1796 and 1939 VACV had replaced CPXV as the smallpox vaccine. This is likely to have occurred in the 19th century because late 19th century pathologists described the eosinophilic cytoplasmic inclusion bodies caused by the smallpox vaccine (and that are made by both VACV and CPXV) but did not mention the more obvious A type inclusion bodies that are made by CPXV but not VACV. Interestingly, sequence analysis of a smallpox vaccine sample from USA in 1902 indicated that this is most closely related to HSPV [62]. The actual virus used by Jenner in 1796 has often been reviewed but remains uncertain [17,63,64].

VACV, HSPV, CPXV and VARV are all orthopoxviruses and are all antigenically related and cross-protective due to the high degree of amino acid sequence conservation of their structural proteins [18]. Many VACV strains were used during the WHO smallpox eradication campaign and, prior to vaccination procedures and quality control methods being standardised, the vaccines varied from country to country [11]. The VACV strains Lister, Copenhagen (Cop), chorioallantois vaccinia Ankara (CVA), Tian Tan (TT), Bern and New York City Board of Health (NYCBH) are examples of strains used during the eradication programme, known as first-generation smallpox vaccines [11,13,65]. It was recognised that these VACV strains induced different frequencies of vaccine-related complications and consequentially strains such as Bern and Copenhagen were less favoured in comparison to Lister and NYCBH [11,66]. After smallpox had been eradicated, vaccine production switched to the use of embryonated chicken eggs or tissue culture cells to reduce bacterial contamination and allergies to animal proteins [65]. Later some vaccine strains were plaque purified, such as the ACAM-2000 strain derived from Dryvax/NYCBH [67] and are called second-generation smallpox vaccines [13]. ACAM-2000 is currently the only smallpox vaccine licensed by the Food and Drugs Administration (FDA) and available for use in the USA [68].

In addition to switching smallpox vaccine production to use modern good manufacturing practice (GMP), safer vaccine strains were sought that did not cause the troublesome vaccine-related complications. Repeated passage of the first-generation vaccines in either avian or mammalian cultured cells enabled isolation of attenuated viruses, in the same way that many live attenuated virus vaccines were produced, such as those for yellow fever, polio (Sabin), measles, mumps and rubella. These third-generation smallpox vaccines include LC16m8 that was derived from strain Lister by Hashizume and colleagues in Japan [69] and modified vaccinia virus Ankara (MVA) that was derived from strain CVA by Mayr and colleagues in Germany [70]. MVA was obtained after 572 passages of the CVA strain in chicken embryo fibroblasts (CEFs) and this resulted in the loss of nearly 15% of its genome, due to six large deletions and many smaller mutations [71,72]. MVA is unable, or barely able, to replicate in most mammalian cells in culture, including human cells and lacks several immunomodulators and host range genes expressed by other VACV stains [73,74]. MVA is immunogenic and avirulent in animal models, is considered safe for human use, including in immunosuppressed people due to its incapacity to replicate in human cells, [75,76]. Nevertheless, higher virus titres and multiple doses are needed to produce immune response equal to those induced by the original smallpox vaccines [77,78,79]. LC16m8 is less attenuated than MVA and retains the ability to replicate and cause lesions in human vaccinees, although the lesions were smaller than caused by the parental Lister strain [69]. LC16m8 does not express the B5 protein, due to a frameshift mutation [80] and this causes a small plaque phenotype. Notably humans immunised with LC16m8 show poor immune responses to this protein [81], which is an important target for neutralising antibody against the extracellular enveloped form of VACV [82]. Notably, both MVA and LC16m8 have not been shown to prevent smallpox because they were used after smallpox was no longer endemic in the human populations in which they were tested.

A fourth generation of smallpox vaccines are ones engineered to enhance safety and immunogenicity. Strategies have included deleting genes encoding enzymes affecting nucleotide metabolism, proteins that suppress the immune response to infection, or host range factors [13]. For example, strain New York VACV (NYVAC) was derived from the Copenhagen strain after the deletion of 18 ORFs. Twelve of these lie in a segment between the host range genes *C7L* and *K1L*. Two others (*B13R* and *B14R*) are non-functional gene fragments that are an intact ORF in other VACV strains and encode a serine protease inhibitor. Genes *J2R* and *I4L* encode thymidine kinase and the large subunit of ribonucleotide reductase, respectively. Lastly, genes *A26L* and *A56R* encode structural proteins from intracellular mature virions and extracellular enveloped virions, respectively [83]. Like MVA, NYVAC is unable to replicate productively in human cells but unlike MVA, expression of some NYVAC late proteins is inhibited due to a translational block [84]. Although attenuated, NYVAC still induces a potent immune response [85,86].

In the last two decades, our knowledge of VACV immune evasion strategies has increased immensely [87], thereby providing many target genes to engineer to obtain safer and more immunogenic vaccines that elicit a protective immunological memory against orthopoxviruses. The remaining part of this article is devoted to a consideration of these genes and the consequences of deleting them on virus virulence and immunogenicity.

## 4. Immune Response against Virus Infection: Basis for an Efficient Vaccine

Multicellular organisms have sophisticated mechanisms to fight viral infections. Upon sensing of pathogen-associated molecular patterns (PAMPs), pathogen recognition receptors (PRR) are triggered to activate intracellular signalling cascades. PRRs can be located either at the plasma membrane or in endosomes, the cytosol or nucleus. They bind to different PAMPs, such as dsRNA by endosomal Toll-like receptor 3 (TLR3) or cytoplasmic retinoic acid-inducible gene I (RIG-I), or cytoplasmic DNA by DNA-dependent protein kinase (DNA-PK), reviewed by [88,89]. Then, PRRs activate signalling pathways that lead to the activation of transcription factors that translocate to the nucleus to promote gene expression. The transcription factors nuclear factor kappa light-chain enhancer of activated B cells (NF-κB), interferon (IFN) regulatory factor 3 (IRF3) and activator protein (AP)-1 are key to this first line of the innate immune response and stimulate the synthesis of IFNs, cytokines and chemokines in response to sensing infection [90].

After release from producing cells, cytokines, chemokines and IFNs engage their cognate receptors on the cell surface, inducing inflammation that amplifies the immune response and attracts other immune cells to site of infection, reviewed by [91,92]. IFNs are key for the antiviral response through the induction of IFN-stimulated genes (ISGs), which restrict virus infection at multiple levels, reviewed by [93]. The outcome of these processes is to restrain virus replication and/or dissemination and promote the elimination of the infection until immunological memory is developed, reviewed by [94]. This adaptive, or acquired, immune response is antigen-specific, unlike innate immunity that is not specific to individual antigens. Antigen-presenting cells, such as macrophages, dendritic cells (DCs) and B-lymphocytes display virus antigens complexed with class II major histocompatibility complexes (MHCs) on their surfaces to T-lymphocytes [94]. Antibodies mediate neutralisation of virus particles and CD8^+^ cytotoxic T-lymphocytes (CTL) cause lysis of infected cells [94]. Despite its delayed onset, adaptive immunity provides a more rapid response upon repeated encounter with the same virus [94]. Two other important components of the immune system are complement and natural killer (NK) cells. Complement enhances the clearance of virions and virus-infected cells by antibodies and phagocytic cells, reviewed by [95] and NK cells eliminate virus-infected cells before the development of a virus-specific antibody and cytotoxic T-lymphocyte (CTL) response, reviewed by [96].

### Immune Response against Vaccinia Virus Infection

The immune response against VACV infection has been studied after smallpox vaccination in humans and infection in animal models, mainly in the mouse, reviewed by [97]. Of particular interest are the studies that used intradermal or intranasal routes for virus infection because the former resembles vaccination [98] whilst the later mimics natural infections with orthopoxviruses, such as VARV and MPXV [97].

In the mouse intranasal model, VACV infects and replicates in the lung leading to inflammation and tissue damage [99,100]. The virus disseminates to secondary sites, including lymph nodes, brain, liver, kidneys, spleen, gastrointestinal tract and gonads. VACV replication in these organs results in a second wave of viraemia that mimics the progression of some other orthopoxviruses in their hosts, such as ectromelia virus (ECTV) in mice [101] and VARV in humans [11]. Expression of type I IFNs, chemokines and cytokines, such as tumour necrosis factor (TNF)-α and IFN-γ, are upregulated in the lungs and inflammatory cells are recruited, such as macrophages, T-lymphocytes and NK cells [98,102]. Early after infection, innate immune cells, such as macrophages, DCs and NK cells, restrict viral replication in the lungs [102,103,104]. However, to limit VACV dissemination to secondary sites and clear the infection, a VACV-specific CD8^+^ T-cell response is required [105,106,107]. IFN-γ is produced by both NK cells and CD8^+^ T-cells and plays an important role in the resolution of respiratory VACV infection [102,105].

In the intradermal mouse model, VACV replication remains local with little or no spread from the primary site of infection and no signs of systemic disease [108]. After infection of skin, the inflammatory infiltrate recruited to the site of infection is composed mainly of macrophages, neutrophils and T-cells [98,109]. Initially, macrophages control virus replication and tissue damage locally [110]. Later during the adaptive phase, T-cells control the infection at the inoculation site and provide help for B-cells to produce antibodies, which, in conjunction with T-cells, prevent viral dissemination and promote virus clearance [111].

Studies with animal models have aided understanding of the components of the adaptive immune system that provide protection against secondary orthopoxvirus infection and, therefore, should be induced by vaccination. Whilst during primary infection, T-cells are sufficient for virus clearance before antibodies can be detected, in mice immunised with replication-competent or replication-deficient VACV strains, an antibody response can protect against both disease and mortality, with CD8^+^ T-cells playing a role only in the absence of antibody [112,113,114]. NK cells can also mediate protection against challenge in VACV-vaccinated mice in the absence of an adaptive T-cell response, demonstrating that these cells too can have pathogen-specific memory [115]. Investigation of the immune response elicited after smallpox vaccination and after natural orthopoxvirus infection in humans, also provides valuable information that is useful for the design of more effective VACV-based vaccines [82,116,117,118].

## 5. Vaccine Engineering by Targeting Vaccinia Virus Proteins That Inhibit the Immune Response

To enter their host, replicate and be transmitted to new hosts, mammalian viruses must overcome the hostile environment presented by the innate and adaptive immune system. Consequently, during co-evolution with their hosts, viruses have acquired numerous countermeasures to evade or suppress the immune response. Orthopoxviruses exemplify this well and encode scores of proteins devoted to evasion of innate immunity. VACV is the most intensively studied orthopoxvirus and between one third and one half of its approximately 200 genes are non-essential for replication but encode proteins that affect host range, virulence or immune evasion [12,87].

From here on, the virulence, immunogenicity and protective efficacy of VACV strains engineered to lack the expression of specific immunomodulatory or host range proteins is considered. Because VACV encodes dozens of proteins that affect the outcome of infection in vivo, we focus primarily on studies that tested the protective efficacy of VACV mutants lacking specific immunomodulators against an orthopoxvirus challenge in the appropriate animal model. Engineered VACV strains are also being tested as vaccine candidates against other infectious diseases, such as AIDS, tuberculosis and malaria [65]. Most of the studies described here used VACV strain Western Reserve (WR), which is not a human vaccine strain and was derived from the NYCBH vaccine strain by passage in the brain of new-born mice [119]. Other studies investigated the effects of deleting immunomodulatory genes from human vaccine strains such as MVA, Copenhagen, Lister, NYCBH and NYVAC.

### 5.1. Host Range Proteins

VACV exhibits a broad host range both in cell culture and in vivo, infecting many mammalian species, such as rodents, lagomorphs, ungulates, non-human primates and humans [45,97,120]. The restricted host range of VACV strains lacking specific genes, such as genes *K1L*, *C7L* and *E3L*, identified VACV host range proteins and the properties of these, and host range proteins from other poxviruses, were reviewed [121]. Replication of VACV mutants engineered to lack *E3L* (vΔE3) or *C7L* and *K1L* (vΔC7ΔK1) is abortive in most mammalian cell lines [122,123]. The C7, E3 or K1 proteins antagonise host antiviral factors, such as the ISG products, or prevent programmed cell death before the virus has completed its replication cycle [124,125,126,127]. Mutants lacking these genes are attenuated and their potential as vaccines has been investigated (Table 1).

Despite lacking amino acid sequence similarity with each other, either C7 or K1 can restore replication of vΔC7ΔK1 in most mammalian cell lines by antagonising the effects of type I IFN [125] and the antiviral effects of ISG products interferon regulatory factor (IRF)1 and SAMD9 [136,137]. K1 somehow blocks the activation of protein kinase R (PKR) by viral dsRNA, which leads to activation of NF-κB and inhibition of host protein synthesis, even in presence of C7 [138,139]. K1 also antagonises the activation of NF-κB by preventing the degradation of the inhibitor of κBα (IκBα) and, further downstream in the pathway, by inhibiting the acetylation of p65 that is required for NF-κB activation [140,141]. VACV strain MVA lacks the *K1L* gene but still encodes C7, which is essential for late viral gene expression in human and murine cells [124].

A VACV WR mutant lacking K1 (vΔK1) replicated normally in cell culture but was attenuated in both intradermal and intranasal mouse models of infection [134]. Infection via the intranasal route induced less weight loss and lower virus titres compared to control viruses in primary (lungs) and secondary (spleen, brain) organs [134]. Despite producing smaller dermal lesions, vΔK1 replicated as well as wild-type and revertant controls and led to decreased immune cell infiltration and inflammatory gene expression. Nonetheless, compared to control viruses, intradermal infection with vΔK1 elicited the same levels of VACV-specific CD8^+^ T-cells and conferred the same protection against challenge with VACV WR [134]. The comparable immunological memory induced by vΔK1, despite the muted local inflammatory response, offers an opportunity to study the importance of the inflammatory response elicited by an attenuated vaccine for its protective efficacy [142,143,144].

Genes *C7L* and *K1L* are amongst the 18 ORFs deleted from VACV strain Copenhagen to form strain NYVAC [83], which does not replicate in human cells due to translational block and induction of apoptosis [84]. Re-introduction of C7 in NYVAC restored growth in human cells but the virus virulence remained unaltered [84]. Although the protective efficacy of NYVAC-C7 against an orthopoxvirus challenge has not been tested, it has shown promise as a candidate vaccine against other diseases. Re-insertion of gene *C7L* into NYVAC expressing four clade B HIV-1 antigens (env, gag, pol and nef) (NYVAC-B-C7) elicited a stronger T-cell response to the HIV-1 antigens than did NYVAC-B in a DNA prime/virus boost regimen in mice [126]. Also, NYVAC-C7 expressing *Leishmania* activated C-kinase (LACK) elicited a stronger LACK-specific T-cell responses and was more protective than the parental control vector against a *Leishmania infantum* intradermal challenge in a DNA prime/virus boost regimen tested in mice [145].

Deletion of both *C7L* and *K1L* from VACV strain Tian Tan (VTTΔC7ΔK1) induced greater attenuation than single-deletion mutants (VTTΔC7 and VTTΔK1) in mice (intracranial and intranasal) and rabbits (intradermal) [135]. Intramuscular vaccination of mice with VTTΔC7ΔK1, induced a lower antibody response whilst the T-cell response remained unchanged [135]. Future research might study how the virulence and immunogenicity of *C7L* and *K1L* single- and double-deletion mutants compare with other VACV strains and how well these mutants confer protection against an orthopoxvirus challenge.

Deletion of gene *E3L* led to abortive replication in most mammalian cell lines [122]. E3 is a multifunctional protein with an N-terminal Z-DNA-binding domain and a C-terminal RNA-binding domain, both of which are required for virulence [146,147,148]. The C-terminal domain binds dsRNA and prevents the activation of PKR and 2′5′-oligoadenylate synthase (OAS) [146,147,148,149,150,151]. E3 also counteracts the antiviral activities of another ISG, ISG15 [152]. Through its RNA-binding domain, E3 also blocks the sensing of RNA derived from the transcription of AT-rich DNA by RNA polymerase III [153,154]. The Z-DNA-binding domain prevents virus-induced necroptosis triggered via DNA-dependent activator of IFN-regulatory factors (DAI) in IFN-treated cells [155]. Engagement of PKR by dsRNA leads to activation of MAPK, NF-κB and IRF3, hence inhibition of PKR by E3 also interferes with the stimulation of these three immune signalling pathways [156,157,158]. However, E3 inhibits NF-κB and IRF3 activation independently of PKR [158,159]. In MVA, expression of E3 allows production of viral intermediate and late proteins in human and murine cells [160].

A VACV WR strain lacking E3 expression (vΔE3) is highly attenuated in vivo in normal and immunodeficient mice infected via intranasal route or tail scarification; vΔE3 is also attenuated when inoculated intracranially in immunocompetent mice [128,129]. Unlike WT virus, replication of vΔE3 was limited to primary sites of infection (noses in the intranasal model, or skin in the tail scarification model) and no dissemination to secondary sites was detected [128,129]. Gene *E3L* deletion from NYCBH (NYCBHΔE3) and Copenhagen (vCopΔE3) caused attenuation in the murine intranasal model [129] but immunisation via intranasal route or tail scarification protected mice against VACV challenge [128,129]. Tail scarification induced a strong VACV-specific T-cell response and immunological protection even in B-cell-deficient mice, highlighting that antibodies are not essential for protection against VACV infection in mice [128].

NYCBHΔE3 vaccination by scarification was protective in alternative models as well, i.e., rabbitpox virus (RPXV) infection in rabbits, ECTV in mice and MPXV in cynomolgus macaques. Whilst in mice, vaccination with one or two doses of NYCBHΔE3 protected against a ECTV challenge, in rabbits, two doses of NYCBHΔE3 were necessary to protect against high-dose RPXV challenge in comparison to a single dose of NYCBH [130,131]. In rabbits, vaccination with NYCBHΔE3 did not prevent development of secondary lesions in RPXV-challenged animals, though two doses of NYCBHΔE3 were as protective as one dose of NYCBH and both strains induced comparable titres of anti-VACV neutralising antibodies [130]. In cynomolgus macaques, vaccination with two doses of NYCBHΔE3 conferred 75% protection against a MPXV challenge compared to 100% protection conferred by a single dose of NYCBH [132]. Moreover, as observed in RPXV-challenged rabbits, NYCBHΔE3 did not prevent viremia and development of secondary lesions in MPXV-challenged animals, whereas NYCBH did [132]. Similar results were observed when *E3L* was deleted from MVA. Despite producing comparable VACV-specific CD8^+^ T-cell responses, mice immunised with the resulting virus (MVAΔE3) were less protected against a lethal ECTV challenge than mice immunised with MVA [133]. Altogether, these results suggest that attenuation of vaccine strains by deletion of *E3L* might fail to produce a sufficiently immunogenic and protective vaccine, probably because of the severe replication defects exhibited by such deletion mutants.

### 5.2. Secreted Proteins

Some of the VACV immunoevasins are secreted from the infected cell and bind and inhibit cytokines, chemokines, IFNs and complement factors extracellularly (Table 2).

#### 5.2.1. Protein C21: Vaccinia Complement Control Protein—VCP

VACV complement control protein (VCP, gene *C21L*) is related to complement control proteins [176] and binds to C3b and C4b to block complement activation by either the classical or alternative pathway [174,177,178]. VCP is not needed for replication in cell culture [177] but a deletion mutant, vΔVCP, was attenuated in rabbits [174] and mice [175]. vΔVCP infection was associated with increased infiltration of CD4^+^ and CD8^+^ T-cells, reduced local virus titres and increased VACV-specific antibodies and all these differences were reversed to wild-type levels in complement-deficient C3^−/−^ mice [175]. Also, only concomitant depletion of CD4^+^ and CD8^+^ T-cells reversed lesion size and viral titres in vΔVCP-infected mice, showing that protection against VACV infection in the intradermal mouse model is mediated both by CD4^+^ T-cell- (i.e., antibody-dependent) and CD8^+^ T-cell-dependent mechanisms [175]. Intradermal infection with vΔVCP induced better protection than wild-type against VACV challenge [175].

#### 5.2.2. Protein A41: A Secreted Chemokine-Binding Protein

A41 is another secreted protein encoded by VACV [161]. A41 binds CC chemokines CCL21, CCL25, CCL26 and CCL28 and prevents their interaction with glycosaminoglycans and the formation of a chemokine concentration gradient that is important for the recruitment of leukocytes [179]. A VACV WR lacking gene *A41L* (vΔA41) replicated normally in cell culture but had slightly increased virulence in the murine intranasal model and produced larger lesions after intradermal infection of mouse ear pinnae and increased infiltration into infected rabbit skin [161,162]. Despite increased lesion size, vΔA41-infected mice cleared the infection more rapidly than controls [161]. In the murine intranasal model, no difference in the viral titres and in the pulmonary CD8^+^ T-cell response was observed with vΔA41. Nevertheless, vΔA41 caused an increase in the VACV-specific splenic CD8^+^ T-cell response compared to mice infected with control viruses [162]. The VACV-specific CD8^+^ T-cell response also increased in the spleens of mice immunised by a prime-boost regimen with an MVA strain lacking A41 (MVAΔA41). More importantly, MVAΔA41 immunisation conferred better protection against VACV challenge, suggesting it is a more potent vaccine [162].

#### 5.2.3. vCCI: A Secreted CC Chemokine-Binding Protein

VACV protein A41 is related to another CC-chemokine-binding protein expressed by some VACV strains (e.g., Lister and RPXV) but not by others (e.g., Copenhagen and WR). This protein is called VACV chemokine-binding protein (vCKBP) or VACV CC chemokine inhibitor (vCCI) [180,181,182,183]. Soluble vCCI binds with high affinity to CC (RANTES, MIP-1α, MCP-1, eotaxin) but not CXC (IL-8, GROα, ENA-78) or C (lymphotoxin) chemokines, thereby blocking their interaction with cellular receptors [180]. The impact of vCCI expression on VACV virulence was investigated with a VACV WR strain engineered to express Lister vCCI from the *B8R* gene locus (vΔB8-ListervCCI) [170]. The absence of protein B8 did not affect virulence in murine models of infection [163,171]. vΔB8-ListervCCI induced reduced weight loss, signs of disease and mortality after intranasal infection in comparison to vΔB8, indicating that vCCI expression attenuated VACV virulence [170]. Attenuation due to vCCI expression was associated with lower viral titres and viremia, reduced pulmonary recruitment of leukocytes (mainly macrophages and lymphocytes) and consequently decreased inflammation and oedema [170]. The impact of vCCI on immunogenicity needs to be investigated.

#### 5.2.4. C12: A Soluble IL-18-Binding Protein

VACV encodes several secreted, cytokine-binding proteins. One example is C12, an early, non-essential protein [167]. C12 binds interleukin (IL)-18 [167,184,185] and blocks IL-12-induced production of IFN-γ [167], which acts in synergy with IL-12 to regulate the immune response. A virus lacking the *C12L* gene (vΔC12) was attenuated after intranasal infection of mice [167] and there was increased IL-18 and enhanced NK-cell cytotoxicity early at 3 days post-infection (dpi) in the lungs. Later, at 7 dpi, IL-18 led to increased production of IFN-γ and nitrite and enhanced CTL response in the lungs of vΔC12-infected mice. C12 therefore counteracted the effects of IL-18 in both innate and adaptive immune responses against VACV infection [186]. Deletion of *C12L* also attenuated VTT, which formed smaller lesions in rabbits infected intradermally and had a lower LD_50_ in the intracranial model of infection in mice [168]. An MVA strain lacking C12 expression (MVAΔC12) elicited a stronger immune response than wild-type control in mice, independently of the immunisation route [169]. Furthermore, mice immunised intraperitoneally with MVAΔC12 lost less weight and had reduced signs of illness then MVA-immunised mice after challenge with VACV WR [169]. This protection correlated with a stronger VACV-specific CD8^+^ T-cell response. Loss of C12 also enhanced the immune response to HIV antigens expressed by MVAΔC12 [169].

#### 5.2.5. Protein B15: A Soluble IL-1β Receptor

VACV WR protein B15 (IL-1β receptor) binds and inhibits human and mouse IL-1β but not IL-1α or the IL-1 receptor antagonist protein [99]. B15 is expressed by several VACV strains but not Copenhagen and is non-essential for VACV WR replication [99]. VACV WR vΔB15 was more virulent in mice infected intranasally at high doses [99]. In contrast, lack of B15 resulted in reduced virulence after intracranial infection of mice [164]. Intranasal infection with vΔB15 induced a febrile response, unlike control viruses, indicating that IL-1β is controlling the induction of fever [187]. Deletion of the MVA counterpart of B15 did not affected MVA replication in CEFs but the deletion mutant MVAΔ184 induced better protection against intranasal challenge with VACV WR, even six months after vaccination, due to enhanced CD8^+^ T-cell memory [165]. Another study with MVA also reported enhanced CD8^+^ T-cell responses followed knock out of this gene [166].

#### 5.2.6. Proteins B8 and B18: IFN-Binding Proteins

VACV strain WR proteins B8 and B18 encode secreted proteins that bind IFN-γ or type I IFNs, respectively. Protein B8 has similarity to the mouse and human IFN-γ receptor (IFN-γR) and to protein M-T7 from myxoma virus [188] and acts as a soluble antagonist of IFN-γ [189,190]. Unlike cellular IFN-γRs that bind IFN-γ mostly from the same species, B8 bound human, bovine, rat and rabbit IFN-γ with high affinity and to mouse IFN-γ with low affinity [189,190]. B8 functions as a homodimer [191] and also binds equine IFN-γ [171]. A VACV WR strain lacking gene *B8R* had normal virulence in mice but was attenuated in rabbit skin [171,192]. Another study found reduced virulence in mice without reduction in humoral immunity [172].

VACV WR protein B18 is an extracellular inhibitor of type I IFNs [173,193] and a member of the immunoglobulin superfamily [194]. Like B8, B18 inhibits IFNs from many species (human, rabbit, bovine, rat and mouse) and is a virulence factor in mouse models of infection [173]. B18 binds to glycosaminoglycans on the surface of cells [195] and so inhibits type I IFNs on the cell surface and in solution [196]. Deletion of *B8R*, *B18R* or both genes, from a NYVAC strain engineered to expresses HIV-1 clade C Env, Gag, Pol and Nef antigens (NYVAC-C) improved HIV-1-specific CD8^+^ T-cell responses [197].

#### 5.2.7. Other VACV Secreted Proteins

Several other VACV proteins are secreted from infected cells but are not considered further here because their influence on the immune response to infection and protection from challenge has not been studied in detail. These include A53, a secreted TNF-α receptor made by VACV strains Lister, USSR, Evans and Tian Tan, also called cytokine response modifier (crm)E [168,198,199]; protein C11, the VACV epidermal growth factor (VGF) responsible for the dermal hyperplasia characteristic of orthopoxvirus infections [200,201]; and protein A39 a secreted glycoprotein with similarity to semaphorins that influences virus virulence and the immune response to infection [202,203].

### 5.3. Intracellular Immunomodulators

The majority of the VACV immunomodulatory proteins act intracellularly to block innate immune signalling pathways that culminate in the production of IFNs, cytokines and chemokines, or the induction of programmed cell death. For instance, VACV encodes at least ten proteins that inhibit NF-κB activation intracellularly (A46, A49, A52, B14, C4, E3, K1, K7, M2 and N1) and six that inhibit IRF3 activation (A46, C6, C16, E3, K7 and N2), for review see [87]. Several of these proteins have multiple functions. These proteins are considered in 2 groups: those with similarity to Bcl-2 (Table 3) and those lacking such similarity (Table 4).

#### 5.3.1. VACV Bcl-2-Like Proteins

VACV encodes a family of proteins with similarity to B-cell lymphoma (Bcl)-2 [225]. Some of these proteins were noted to share limited (~20%) amino acid (aa) identity [226,227] but their relatedness to Bcl-2 proteins was only revealed after the structures were solved by X-ray crystallography, such as N1, B14, A52 and F1 [228,229,230,231]. Other VACV proteins shown to have a Bcl-2 fold are A46 [232], A49 [233] and K7 [234,235], and proteins C6 [212,236] and N2 [214] are predicted to adopt a Bcl-2 fold. A function for most of these proteins has been reported and several of these proteins have multiple functions.

##### Protein B14: An NF-κB Inhibitor

B14 is a cytosolic, early protein that is non-essential for replication but contributes to virulence and affects the recruitment of leukocytes [204]. B14 inhibits NF-κB activation at the level of the IκBα kinase (IKK) complex by binding to IKKβ and preventing its phosphorylation by upstream kinases and consequently preventing the phosphorylation of IκBα [237]. MVA protein 183 shares 95% aa identity with VACV WR B14 but is unstable due to mutation and does not inhibit NF-κB activation [238]. Using the B14 structure [230], a F130K mutation was found to no longer bind IKKβ or inhibit NF-κB activation [239].

##### Protein N1: An Inhibitor of Apoptosis and NF-κB

Like B14, N1 is an early, non-essential protein that promotes virulence [205,206]. N1 inhibits NF-κB activation, although the exact mechanism is uncertain [207,229,240]. N1 forms a homodimer [206] and its structure [228,229] revealed a Bcl-2 fold with a surface groove through which it binds BH3 peptides from pro-apoptotic Bcl-2 proteins [228,229] and thereby inhibits apoptosis [207]. Mutations that fill the surface groove ablated the ability to inhibit apoptosis, whereas mutation of the hydrophobic dimer interface disrupted dimerization and prevented inhibition of NF-κB [207]. Separation of these functions showed that the ability to inhibit NF-κB activation was important for virulence whereas inhibition of apoptosis was not [207]. The attenuation of a mutant VACV WR lacking the *N1L* gene (vΔN1) was linked to a stronger pulmonary NK-cell and CD8^+^ T-cell response during acute infection [109,241]. Immunisation of mice with vΔN1 or a virus expressing vN1-I6E (an N1 mutant unable to dimerise and inhibit NF-κB activation) induced increased protection against intranasal challenge with VACV WR due to stronger CD8^+^ T-cell memory [208,241].

##### Protein K7: An NF-κB and IRF3 Inhibitor

Protein K7 is another early, non-essential VACV protein that promotes virulence [209]. K7 binds to IL-1 receptor-associated kinase 2 (IRAK2) and TNF receptor-associated factor 6 (TRAF6) to inhibit the NF-κB pathway [235,242]. Additionally, K7 prevents IRF3 activation by interacting with the DEAD-box RNA helicase 3 (DDX3) protein [242]. The structure of K7 revealed a Bcl-2 fold but it lacked a surface groove for binding of BH3 peptides [234,235]. Loss of K7 caused virus attenuation in both dermal and intranasal murine models of infection and analysis of infected lungs showed increased infiltrating T-cells and activated macrophages, as well as higher pulmonary NK cell- and CTL-dependent cytotoxic responses [209]. Immunisation with VACV WR lacking K7 induced better protection against challenge [243]. Similarly removal of the *K7L* gene together with gene *C6L* from VACV strain MVA induced stronger CD8^+^ T cell responses against HIV antigens expressed by this virus [244], although in this study the cause of this change was not analysed by deletion of these genes separately.

##### Protein A52: An NF-κB Inhibitor

VACV WR protein A52 is another inhibitor of NF-κB activation and, like K7, can bind to IRAK2 and TRAF6 [210,245]. A52 is expressed early, is non-essential for virus replication and promotes virulence in the murine intranasal model [210]. The structure of A52 showed it is a Bcl-2 protein [230].

The NYVAC strain encodes A52, B14 and K7 and derivative strains engineered to lack one, two or all three genes were tested as candidate HIV vaccines. This showed that, to different extents, these NYVAC deletion-mutants increased cell infiltration, cytokine and chemokine production and HIV-specific CD8^+^ T-cell and antibody responses after intraperitoneal infection of mice [246]. These results highlight the potential of these proteins as targets for further vaccine engineering.

##### Protein A46: An Inhibitor of IRF3, NF-κB and MAPK Pathways

A46 is another NF-κB antagonist with a Bcl-2 fold [232]. A46 is expressed early during infection and binds to adaptor molecules downstream of Toll-like receptors (TLRs) and therefore inhibits the activation of downstream NF-κB, IRF3 and MAPK pathways [211,245,247,248]. A VACV WR *A46R* deletion mutant is attenuated in the intranasal model of infection compared to control viruses and caused an increased and accelerated pulmonary infiltrate [211]. The potential of vΔA46 as a vaccine is yet to be investigated.

An interesting feature of A46 is an N-terminal region before the Bcl-2 fold and recently this structure was solved and shown to have a novel β-sheet fold that contains a myristic acid binding pocket [249].

##### Protein C6: And IRF3 and JAK-STAT Signalling Inhibitor

C6 is an early, non-essential protein that enhances virulence [212]. C6 inhibits activation of IRF3 and IRF7 by binding to the adaptor proteins needed to activate the upstream kinases TANK-binding kinase 1 (TBK1) and IKKɛ. Consequently, C6 restricts the production of type I IFNs [212]. C6 also inhibits activation of the Janus kinase (JAK)-signal transducer and activator of transcription (STAT) signalling pathway following binding of type I IFN to the type I IFN receptor and so prevents transcription of ISGs containing the IFN-stimulated response element (ISRE) [236]. C6 inhibits this pathway in the nucleus, after IFN-stimulated gene factor 3 (ISGF3) binds to the ISRE element and might mediate this inhibition by its association with STAT2 [236]. Vaccination with vΔC6 induced enhanced CD8^+^ T-cell responses and better protection against VACV challenge [213]. Deletion of gene *C6L* from MVA also increased the both humoral and CD4^+^ and CD8^+^ T-cell memory responses against HIV-1 antigens expressed by MVA [250].

Since the deletion of genes *N1L*, *C6L* and *K7R* individually caused increased immunogenicity and decreased virulence, another study investigated whether deleting these genes in combination would improve immunogenicity further. In vivo the attenuation caused by deletion of each single gene was increased stepwise by deletion of the second and third gene in both intranasal and intradermal mouse models [243]. However, although intradermal vaccination with vΔN1 induced better protection against challenge [208], deletion of *N1L* and *K7R* together, or all three genes resulted in inferior protection that correlated with a decrease in CD8^+^ T-cell response and in neutralising antibody titre [243]. Further studies are needed to investigate if deletion of multiple genes in other VACV strains, such as the MVA, gives a similar outcome. In some cases, the deletion of multiple immunomodulators from MVA increased immunogenicity [244,251], however, in another study, an MVA strain lacking fifteen immunomodulatory genes induced similar CD8^+^ T-cell response against both VACV-specific or heterologous antigen peptides [252]. Collectively, these studies highlight that a balance between attenuation and immunogenicity is important in the design of future vaccines.

##### Protein N2: An IRF3 Inhibitor

N2 is an early, nuclear, non-essential protein with a predicted Bcl-2 fold that inhibits IRF3 activation by an unknown mechanism [214]. N2 is highly conserved in VACV strains but in MVA it lacks residues 31–35. VACV WR lacking N2 was less virulent and induced an increased pulmonary cell infiltration but nonetheless induced similar protection to challenge compared to control [214]. However, a MVA lacking N2 and expressing clade B HIV-1 antigens (MVA-BΔN2) elicited greater HIV-1-specific CD4^+^ and CD8^+^ T-cell responses and greater HIV-specific antibodies, although anti-VACV-specific antibodies remained unchanged [253].

##### Protein F1: An Inhibitor of Apoptosis and the Inflammasome

Protein F1 is expressed early and is a potent inhibitor of apoptosis [254,255]. F1 has a Bcl-2-like fold with a surface groove for binding BH3 peptides [231]. F1 also binds and inhibits caspase 9 [256] and blocks inflammasome activation by binding the NOD-like receptors (NLR) family member NACHT, LRR and PYD domains-containing protein 1 (NLRP1) [215]. Mutational analysis of F1 separated inhibition of the inflammasome from inhibition of apoptosis and in vivo analysis showed that inhibition of the inflammasome was important for virus virulence [215]. Deletion of *F1L* from MVA reduced its protective capacity against a lethal ECTV challenge in mice, without affecting virus-specific CD8^+^ T-cell responses [133]. Nevertheless, deletion of *F1L* from an MVA strain expressing Env and Gag-Pol-Nef antigens from clade C HIV-1 (MVA-C-DeltaF1L) enhanced HIV-1-specific CD8^+^ T cell adaptive immune responses following a prime-boost immunisation programme [257].

#### 5.3.2. Proteins C4 and C16

VACV WR proteins C4 and C16 share 43% aa identity and are early, non-essential proteins that promote virulence [216,217]. C16 inhibits DNA sensing leading to IRF3-dependent innate immunity by binding to the Ku proteins Ku70 and Ku80 [258], which are subunits of the DNA-PK complex that functions as a DNA sensor [259]. C16 binds the Ku proteins via its C-terminal domain, whereas the N-terminal region binds the oxygen sensor prolylhydroxylase domain-containing protein 2 (PHD2). The latter interaction prevents PHD2 hydroxylating hypoxia-inducible transcription factor (HIF)-1α and the subsequent ubiquitylation and degradation of HIF-1α. Thus HIF-1α is stabilised, migrates to the nucleus and induces transcription of genes bearing the HIF-responsive element leading to a hypoxic response [260]. In turn, this leads to re-programming of central energy metabolism with a switch from oxidative phosphorylation to glycolysis [261]. C16 is conserved in several VACV strains, including WR, Copenhagen, Lister and MVA [217], although an internal 5-aa deletion in MVA C16 greatly reduces binding to Ku [258]. Mice infected intranasally with vΔC16 showed a faster pulmonary recruitment and activation of CD4^+^ and CD8^+^ T-cells [217]. The effect of C16 on immunological memory has not been reported.

Protein C4 inhibits NF-κB activation at or downstream of the IKK complex [216]. Like C16, C4 affected virulence in the intranasal but not intradermal, mouse model and its deletion caused increased pulmonary infiltration of leucocytes [216]. A comparison of the conservation of C4 and C16 amongst orthopoxviruses showed that few viruses expressed both proteins but all express either one or the other, suggesting a conserved and important function [216,217]. It will be interesting to investigate the in vivo phenotype of a VACV strain lacking both C16 and C4 and the effects on the induction of immunological memory.

#### 5.3.3. De-Capping Enzymes D9 and D10

VACV also blocks activation of host immune responses by targeting protein translation. D9 and D10 are de-capping enzymes expressed either early (D9) or late (D10) during infection [262,263]. Both enzymes remove the cap from cellular and viral mRNAs and are important for shutting off host protein synthesis and for enhancing turnover of viral mRNAs and thereby the transition from the early to intermediate to late phases of virus protein expression [262,263]. D9 and D10 also cooperate to prevent the formation of dsRNA in infected cells, thereby blocking the activation of PKR [218]. The absence of a functional D10 enzyme but not D9, caused virus attenuation in mice in both intranasal and intraperitoneal models of infection [218]. However, when both D9 and D10 had inactivating mutations in their catalytic sites, the mutant VACV exhibited a replication defect in cell culture and severe attenuation in vivo [218]. Although highly attenuated, this double-mutant virus protected mice against challenge with VACV WR [218].

#### 5.3.4. Protein 169: An Inhibitor of Translation

Protein 169 is an early, small, charged, intracellular non-essential protein that suppresses the immune response by inhibition of cap-dependent and cap-independent translation initiation [219]. A VACV WR mutant lacking 169 expression (vΔ169) is more virulent in infected mice, causing larger lesions after intradermal infection or greater weight loss after intranasal infection [219]. Mice immunised with vΔ169 were better protected against challenge with VACV than mice infected with control viruses [219]. Early after infection with vΔ169 there was increased pulmonary cell infiltration and increased production of inflammatory cytokines and chemokines. Later there was increased macrophage and T-cell (CD4^+^ and CD8^+^) infiltration in the lungs but no difference in neutralising antibodies levels [219].

#### 5.3.5. Protein A35

Protein A35 is an intracellular, hydrophobic protein that localises to virus factories and contributes to virulence [220]. A35 restricts antigen presentation to T-cells via MHC class II. Infection with a virus lacking A35 (vΔA35) induced lower VACV-specific antibodies and IFN-γ secretion and lysis by splenocytes [221,264]. Despite being attenuated, vΔA35 conferred equivalent protection against challenge [221]. Immunisation with MVA lacking *A35R* (MVAΔA35) also induced protection as well as wild-type MVA [222]. MVAΔA35 induced greater VACV-specific antibodies, an increased percentage of granulocytes, CD11b^+^ cells and IFN-γ-producing cells in spleen but no difference in the percentage of CD8^+^ T-cells [222]. Deletion of *A35R* from an L-IPV strain lacking genes *C3L*, *N1L*, *J2R*, *A56R* and *B8R*, induced greater VACV-neutralising antibody when used in prime-boost regimen and equivalent protection against ECTV challenge [265].

#### 5.3.6. Protein A44: A 3β-Hydroxysteroid Dehydrogenase

Protein A44 shares 31% aa identity with human 3β-hydroxysteroid dehydrogenase (3β-HSD), an enzyme needed for steroid biosynthesis [226,227,266]. Several other poxviruses such as molluscum contagiosum virus [267], fish lymphocystis virus [268] and avipoxviruses (fowlpox virus and canarypox virus) [269] also encode 3β-HSD. VACV A44 is an active 3β-HSD and is an early, non-essential protein that promotes virulence [163,223,224]. *VACV* WR lacking 3β-HSD caused reduced corticosterone in lungs and plasma and an enhanced inflammatory response. There were increased IFN-γ levels, more rapid recruitment of CD4^+^ and CD8^+^ T-cells and a stronger cytolytic T-cell response to VACV-infected cells [270]. Immunisation of mice with VACV strain Praha or Dryvax derivatives lacking 3β-HSD and expressing either varicella-zoster virus glycoprotein E or hepatitis B virus glycoprotein pre-S2 showed either no difference in antibody levels to VACV or the foreign antigens, or a slight reduction, depending on the parental strain used [224].

#### 5.3.7. Other Intracellular Virulence Factors or Immunomodulators

Many other VACV proteins, either immunomodulators or proteins with other functions, affect virulence and/or the host immune response. These include I4 (ribonucleotide reductase large subunit) [271,272], J2 (thymidine kinase) [273], A26 (virion attachment/entry) [274], B5 (virion envelope glycoprotein related to complement control factor) [275,276], B13 and B22 (serine protease inhibitors SPI-2 and SPI-1, respectively) [163,277,278,279], F12 (IEV transport protein) [280], A36 (IEV transport protein and actin tail inducer) [163,281], A40 (cell surface glycoprotein) [163,282], A50 (DNA ligase) [283], C2, F3 and A55 (intracellular kelch protein immunomodulators) [284,285,286] and A33 (virion envelope glycoprotein) [276], However, the protective capacity of viruses lacking these proteins requires further study.

### 5.4. Expression of Cellular Immunomodulators from Vaccinia Virus

An alternative strategy to improve safety and/or immunogenicity of VACV vaccine is the expression of cellular immunomodulators from the virus. This strategy has been tested with recombinant VACV strains expressing cytokines such as IL-2 [287,288], IL-4 [289], IL-18 [290], IFN-γ [291,292,293], IFN-λ2 and IFN-λ3 [294]. Whereas expression of IL-4 from VACV delayed virus clearance in mice, VACV expressing IL-2, IL-18, IFN-γ or IFN-λ are attenuated in vivo. Also, expression of chemokine CCL20 or colony-stimulating factor GM-CSF from MVA induces a stronger virus-specific CD8^+^ T-cell and antibody responses after immunisation of mice [295].

## 6. Conclusions

Safer and immunogenic vaccines against orthopoxviruses are needed to combat emerging orthopoxvirus infections in Europe, South America and India, monkeypox in Africa and the possible re-creation of orthopoxviruses by synthetic biology [20]. The VACV genome encodes scores of immunomodulatory proteins, some of which remain poorly or incompletely characterised [87]. Many VACV mutants engineered to lack these immunomodulatory proteins display an altered virulence and/or immune responses, showing that engineering safer and more potent VACV vaccines is possible [87]. Although the use of attenuated replication-deficient strains, like NYVAC and MVA, is desirable for use in those with immunodeficiency [76], immunisation with NYVAC and MVA requires higher or multiple doses to confer the same protection as replication-competent strains [112].

Deletion of some immunomodulatory genes, for examples *A41L* and *A35R*, has increased the immunogenicity of MVA [162,222]. On the other hand, deleting the host range gene *E3L* produced inferior vaccines, possibly due to restricted replication of the vaccine, illustrating that there is balance between vaccine replication/attenuation and immunogenicity/protection [130,132]. Further research is needed on proteins whose deletion increases protective efficacy but also the virulence of replication-competent strains, like VACV WR protein 169 [219]. It would be interesting to study the consequence of deleting 169 from replication-deficient strains that encode it, such as MVA, or to restore its expression in strains that lack it, such as NYVAC. The optimal balance between attenuation, immunogenicity and immunisation dose is hard to predict and needs to be evaluated on a case-by-case basis. So far, the deletion of multiple immunomodulatory genes has not given predictable outcomes for immunogenicity in animals [243,265] and how these will affect outcomes in humans is unknown. A systematic analysis of different combination of deleted genes, along with comparison with wild-type, single-deletion and revertant controls, is necessary to work out the role of individual genes and whether this is a good strategy for engineering of VACV vaccines. Synthetic biology might be a useful tool to rapidly construct different combinations of gene deletions from custom-synthesized VACV genomes with desired modifications.

## Figures and Tables

**Table 1:** VACV strains lacking host range proteins that have been tested for virulence and/or immunogenicity.

Protein	Function	Virulence (Mouse)			Virulence (Other Models)		Protective Efficacy/Immune Response	References
		i.d.	i.n.	i.c.	Rabbit (i.d.)	Macaque (i.d.)		
E3	dsRNA-binding protein	↓	↓	↓	↓	↓	↓ protection (WR, NYCBH, MVA)n.c. CD8^+^ T-cell response	[128,129,130,131,132,133]
K1	NF-κB, PKR, IRF1 and type I IFN antagonist	↓	↓				↑ protection (WR)n.c. CD8^+^ T-cell response	[134]
C7	PKR, IRF1 and type I IFN antagonist		n.c.		n.c.			[135]

Virulence: virulence of mutant virus lacking the protein compared to controls in the indicated model. Protective efficacy: strain of VACV used for immunisation shown in brackets. Arrows pointing up or down indicate increase or decrease, respectively, in virulence or protective efficacy. i.d. intradermal; i.n. intranasal; i.c. intracranial; n.c. no change. PKR, protein kinase R; IRF1, interferon regulatory factor 1; NF-κB, nuclear factor kappa light-chain enhancer of activated B cells.

**Table 2:** VACV strains lacking secreted immunomodulators that have been tested for virulence and/or immunogenicity.

Protein	Function	Virulence			Protective Efficacy/Immune Response	References
		i.d.	i.n.	i.c.		
A41	CC-chemokine binding protein	↑	↑		↑ protection (MVA)↑ CD8^+^ T-cell response	[161,162]
B15	IL-1β binding protein	n.c.	↑	↓	↑ protection (MVA)↑ CD8^+^ T-cell response	[99,163,164,165,166]
C12	IL-18 binding protein	↓ (rabbit skin)	↓	↓	↑ protection (MVA)	[98,167,168,169]
vCCI *	CC-chemokine binding protein		↓			[170]
B8	IFN-γ binding protein	↓ (rabbit skin)	n.c./↓	n.c.		[171,172]
B18	type I IFN binding protein		↓			[173]
C21, VCP	complement control protein	↓			↑ protection (WR)	[174,175]

Virulence: virulence of mutant virus lacking the protein compared to controls in indicated mouse model unless stated otherwise. Immune response: changes in immunological memory. Protective efficacy: strain of VACV used for immunisation shown in brackets. * Impact on virulence was done with a VACV WR strain engineered to express Lister vCCI from the *B8R* gene locus (vΔB8-ListervCCI). Arrows pointing up or down indicate increase or decrease, respectively, in virulence, protective efficacy or immune response. i.d. intradermal; i.n. intranasal; i.c. intracranial; n.c. no change.

**Table 3:** VACV strains lacking intracellular Bcl-2-like immunomodulators that have been tested for virulence and/or immunogenicity.

Protein	Function	Virulence			Protective Efficacy/Immune Response	References
		i.d.	i.n.	i.c.		
B14	NF-κB inhibitor	↓	n.c.			[204]
N1	NF-κB and apoptosis inhibitor	↓	↓	↓	↑ protection (WR)↑ CD8^+^ T-cell response	[205,206,207,208]
K7	NF-κB and IRF3 inhibitor	↓	↓		↑ protection (WR)	[209]
A52	NF-κB inhibitor		↓			[210]
A46	NF-κB, IRF3 and MAPK inhibitor		↓			[211]
C6	IRF3 and JAK/STAT inhibitor	↓	↓		↑ protection (WR)↑ CD8^+^ T-cell response	[212,213]
N2	IRF3 inhibitor	↓	↓		n.c. protection (WR)	[214]
F1	apoptosis and inflammasome inhibitor		↓		↓ protection (MVA)n.c. CD8^+^ T-cell response	[133,215]

Virulence: virulence of mutant virus lacking the protein compared to controls in indicated mouse model. Immune response: changes in immunological memory. Protective efficacy: strain of VACV used for immunisation shown in brackets. Arrows pointing up or down indicate increase or decrease, respectively, in virulence, protective efficacy or immune response. i.d. intradermal; i.n. intranasal; i.c. intracranial; n.c. no change.

**Table 4:** VACV strains lacking other intracellular immunomodulators that have been tested for virulence and/or immunogenicity.

Protein	Function	Virulence				Protective Efficacy/Immune Response	References
		i.d.	i.n.	i.c.	i.p.		
C4	NF-κB inhibitor	n.c.	↓				[216]
C16	inhibitor of DNA sensing and promoter of hypoxic response	n.c.	↓			n.c. protection (WR)	[217]
D9	de-capping enzyme		n.c.		n.c.	n.c. protection (WR)	[218]
D10	de-capping enzyme		↓		↓	n.c. protection (WR)	[218]
169	inhibitor of translation	↑	↑			↑ protection (WR)↑ CD8^+^ T-cell response	[219]
A35	inhibitor of MHC class II antigen presentation		↓		↓	↑ protection (WR)n.c. protection (MVA)	[220,221,222]
A44	3β-hydroxysteroid dehydrogenase	↓	↓	↓			[163,223,224]

Virulence: virulence of mutant virus lacking the protein compared to controls in indicated mouse model. Immune response: changes in immunological memory. Protective efficacy: strain of VACV used for immunisation shown in brackets. Arrows pointing up or down indicate increase or decrease, respectively, in virulence, protective efficacy or immune response. i.d. intradermal; i.n. intranasal; i.c. intracranial; i.p. intraperitoneal; n.c. no change.
